# “What is in a name?”—Definition of mental disorder in the last 50 years: a scoping review, according to the perspective of clinical psychology

**DOI:** 10.3389/fpsyg.2025.1545341

**Published:** 2025-06-02

**Authors:** Donatella Rita Petretto, Alessandro Mura, Gian Pietro Carrogu, Luca Gaviano, Riccardo Atzori, Mattia Vacca

**Affiliations:** Department of Education, Philosophy, Psychology, State University of Cagliari, Cagliari, Italy

**Keywords:** mental disorder, definition, clinical psychology, theoretical framework, DSM

## Abstract

**Background:**

Since ancient times, and later in the 17th and 18th centuries, scholars have sought to classify mental disorders. However, it wasn’t until the late 1970s, with the work of Spitzer and Endicott and the publication of the DSM-III, that a formal definition of mental disorder was established. Once this milestone was reached, a long and complex debate on the definition of mental disorder started. Here we aim to review such a debate, taking into consideration the papers that have proposed and/or investigated and discussed the various definitions of mental disorder over the past 50 years.

**Methods:**

We conducted the literature review via Scopus, PubMed, and Web of Science databases. The following inclusion criteria were established: papers on the definition of mental disorder, written in English. All study designs were eligible, including those that utilized qualitative and quantitative methods, as well as methodology or guideline reports.

**Results:**

The sorted papers (*n* = 64) showed a complex, long and still ongoing debate on the definition of mental disorder. Only few authors directly proposed their own definitions, while others analyzed, discussed, criticized and/or proposed integrations to previous or current definitions. The authors of the selected papers conducted their work alongside the development of various editions of the main international diagnostic manuals (primarily the DSM). Many of them directly engaged with past and present definitions of mental disorder provided in the mentioned manuals or referenced different editions of both DSM and ICD.

**Conclusion:**

We concluded that despite the complex and still ongoing debate on the topic, and the precious contributions from all the authors involved, a unique and agreed-upon definition of the concept of mental disorder is still far from being identified. Furthermore, even if the definitions proposed by the international diagnostic manuals (especially by the DSM) constitutes an undeniable landmark, such definitions resulted to be more the outcome of various discussions, than the fruit of a shared consensus. The construct of mental disorder and its shared definition remain a critical theme in psychopathology.

## Introduction

In the study of mental disorders, the efforts to classify different kinds of disorders started well before the efforts to define what a mental disorder actually is. Since ancient times, and then in the 17th and in 18th centuries, various scholars have tried to classify mental disorders, mainly from a medical point of view, and according to different approaches (symptomatological approaches versus causation-based approaches) (Shorter, 2015; Mack et al., 1994). Such scholars also used different levels of categorization of signs and symptoms: some authors regarded specific behaviors or other specific signs or symptoms as indicators of mental disorder (De Sauvages, 1768), while other authors focused instead on the co-occurrence of various signs and symptoms, and/or behavioral, cognitive and affective features, pioneering the use of the “syndrome” construct (like in the work of Sydenham, 1682). In 1785, Colombier and Doublet published “Instruction on Psychiatry” in which they described four highly inclusive categories of mental disorder, namely: “manie,” “phrènèsie,” “melancolie,” and “stupiditè” (Berlinck, 2012). Anyway, it is better to clarify that in those times there wasn’t yet any clear distinction between the idea of “disorder” and the idea of “mental disorder.” Later, in the early 19th century, Pinel (1801) proposed a new method for studying the so-called “mental alienation” based on a deeper study of the observable symptoms. Then, Esquirol (1820, 1838), following Pinel’s teachings, focused on the understanding of the patient through accurate clinical descriptions, rather than through theoretical speculation (Mack et al., 1994). In the late 19th century, Emil Kraepelin proposed the first systematic nosology based on signs and symptoms, according to a descriptive approach, and solely involving external and observable behavioral features: he insisted that his students as well as the other clinicians should not interpret what they see but rather limit themselves to describe it (Kraepelin, 1883; Decker, 2007). Furthermore, Kraepelin’s work represented a significant shift from the diffuse idea that “mental disorders run on individual courses” and that “generalizations are not helpful,” proposing one of the first generalized notions of “insanity” (Brown et al., 2014). He also wrote a psychiatry textbook with a clear classificatory approach, based on etiology and symptomatology, grouping together all the functional psychotic disorders into three large groups (dementia praecox, manic-depressive illness, and paranoia) (Kraepelin, 1883; Decker, 2007). His textbook soon became the foundation of such a classificatory approach and led to the unintended consequence of creating a common language for clinicians. This occurred even though Kraepelin’s primary aim was to bring order into his own observations, rather than to propose a shared diagnostic manual or offer a contribution to the current situation, which was marked by wide differences in terminology and conceptions (Mack et al., 1994; Decker, 2007; Brückner, 2023).

Soon after, an intense debate started between the medical approach and the so-called “biopsychosocial approach” (at that time, such a term was primarily used to refer to the psychoanalytic approach), thanks to the work of Sigmund Freud and other psychoanalysis-oriented clinicians. In fact, these clinicians chose a different approach to defining mental disorders, focusing not only on the description of psychological signs and symptoms but also on the pathogenesis of psychological issues, as well as on their meaning and influence on an individual’s life (Mack et al., 1994; Decker, 2007; Brückner, 2023). Before this perspective shift, classificatory approaches attracted most of the attention, and a debate on the growing need of a common language in psychopathology and of an internationally shared classification of “mental disorders” only began later. A fundamental step toward this direction was made soon after the First World War and, even more, after the Second World War. In this regard, veterans came back with new kinds of psychological signs and symptoms, caused by the severe traumatic experiences to which they were exposed during the wars. Those signs and symptoms were unknown in the previous categorizations of mental disorders, so the Army Psychologists and Psychiatrists worked a lot to better understand those new psychological experiences, with the aim of supporting the veterans. The need for a new classification system able to consider both newer and older disorders became more and more clear, alongside the need for international diagnostic categorizations, with the purpose of facilitating communication and cooperation between all of the clinicians and researchers in the field of psychopathology. After the First World War, the American Medico-Psychological Association (subsequently named American Psychiatric Association) produced a list of 22 disorders to be used in all the mental institutions across the USA (Mack et al., 1994). Then, after the Second World War, the search for a shared classification of mental disorders continued and in 1948, the World Health Organization, assumed the responsibility of the 6th version of the International Classification of Diseases, Mortality, Morbidity and Causes of death (ICD-6th version) (WHO, 1948), which contained a specific section about mental disorders (Mack et al., 1994). In the same years, with the aim of overcoming some of the limitations of the cited section of the ICD-6, the American Psychiatric Association created its own classification: the Diagnostic and Statistical Manual of Mental Disorders (DSM, later called DSM-I) (American Psychiatric Association, 1952). Even if the birth of these new international diagnostic manuals constituted a significant landmark for psychopathology, the first editions of such manuals did not provide any specific definition of mental disorder, thus proposing a classification of mental disorders without better clarifying what a mental disorder actually is in the first place. The first and second edition of the DSM (American Psychiatric Association, 1952, 1968) proposed a classification of mental disorders based on a psychoanalytic approach and described 102 and 182 disorders respectively, without providing any general shared definition of mental disorder. The DSM-I (American Psychiatric Association, 1952) diagnostic categories were based on psychodynamic etiological explanations, according to the “maladjustment model” by Meyer (1904, 1908, 1912), and were divided in two main groups of mental disorders: conditions caused by organic brain dysfunction and conditions presumed to be the result of socio-environmental stressors and/or of one’s inability to adapt to such pressures (Kawa and Giordano, 2012). In DSM-I (American Psychiatric Association, 1952), each disorder was described in a relatively short paragraph describing its features and such descriptions were sometimes provided in a vague fashion, with no actual symptomatic criteria for diagnosis (Pierre, 2010).

The DSM-II (American Psychiatric Association, 1968) was again based on the psychodynamic tradition but some changes were made: the group of mental disorders included in the classification was increased to be more inclusive of milder conditions seen in the general population; there were an increased specificity of categorization, thanks to the general tendency to create multiple subdivisions of disorder categories; last but not least, the psychodynamic term “reaction” was removed, to avoid inferences regarding the nature and/or causes of disorders, if not yet known. Furthermore, mental conditions were grouped in “organic brain syndromes,” “psychosis not attributed to physical conditions,” “neuroses,” “personality disorders,” and other conditions (Kawa and Giordano, 2012; Pierre, 2010; Brown et al., 2014). Then, in the same years during which the third edition of the DSM-III (American Psychiatric Association, 1980) was being developed, the debate on mental disorders became even more pronounced, with a significant shift from categorization to definition. Robert Spitzer and Jean Endicott proposed an initial definition of mental disorder in the text “Critical Issues in Psychiatric Diagnosis” (Spitzer and Endicott, 1978). The cited definition portrayed mental disorders as a subset of medical disorders; as they stated: *“a medical disorder is a relatively distinct condition resulting from an organismic dysfunction which in its fully developed or extreme form is directly and intrinsically associated with distress, disability, or certain other types of disadvantages. The disadvantage may be of a physical, perceptual, sexual, or interpersonal nature. Implicitly there is a call for action on the part of the person who has the condition, the medical or its allied professions, and society. A mental disorder is a medical disorder whose manifestations are primarily signs or symptoms of a psychological (behavioral) nature, or if physical, can be understood only using psychological concepts”* (Spitzer and Endicott, 1978, p. 18).

The quoted definition attracted much attention as well as critiques and controversies, mainly related to the so-called “jurisdiction problem.” The main controversies happened between the American Psychiatric Association and the American Psychological Association, which was concerned about the problems raised by including a statement about considering mental disorders a subset of medical disorders in the third edition of the DSM. In fact, if mental disorders are medical disorders does that mean that such disorders should be treated by physicians and/or psychiatrists only? This controversy ultimately led the American Psychiatric Association to abandon any reference to mental disorder being a medical disorder in the final draft of DSM-III (Follette and Houts, 1996). When Robert Spitzer became involved in the Revision Committee of the DSM-II (American Psychiatric Association, 1968) and was later nominated head of the DSM-III Task Force, the first definition of mental disorder finally appeared as it follows: *“each of the mental disorders is conceptualized as a clinically significant behavioral or psychological syndrome or pattern that occurs in an individual and that is typically associated with either a painful symptom (distress) or impairment in one or more important areas of functioning (disability). In addition, there is an inference that there is a behavioral, psychological, or biological dysfunction, and that the disturbance is not only in the relationship between the individual and society. (When the disturbance is limited to a conflict between an individual and society, this may represent social deviance, which may or may not be commendable, but is not by itself a mental disorder)”* (American Psychiatric Association, 1980, p. 6).

Another significant conceptual shift that took place during the same years in which the manuals’ definition of mental disorder was being developed, regarded the debate on the need to achieve shared, valid, and reliable diagnostic criteria for research. In this regard, Robins and Guze (1970), and later, Feighner (1972), the so-called “neokraepelinians,” proposed and discussed a method for increasing the diagnostic validity of mental disorders, based on five main phases: clinical description, laboratory study, exclusion of other disorders, follow-up study, and family study. The authors highlighted the need for systematic studies and for a systematic approach to diagnostic schemes, in order to avoid low diagnostic validity, and, as a consequence, the disrepute of diagnostic classification between both clinicians and researchers, and lay public. Spitzer et al. (1978), with Williams (1982) and the work of other colleagues, referring to the so-called “Feighner’s criteria,” developed their Research Diagnostic Criteria aiming at increasing the reliability of the diagnostic criteria for specific mental disorders. Spitzer’s work had a significant impact during the transition from DSM-II to DSM-III and also in subsequent phases (Decker, 2007).

In the very same years, some authors who skeptically discussed the concept of mental disorder contributed to the debate, proposing very radical stances such as: “mental disorder does not exist” and “mental illness is a myth” (Szasz, 1960; Szasz, 1974; Foucault, 1961), and through some specific experiences on the validity of psychiatric diagnosis, such in the case of the highly discussed and controversial work of Rosenhan (1973).

Soon after the publication of the DSM-III (American Psychiatric Association, 1980), and the spread of the first formal definition of mental disorder, a long and still ongoing debate started. In 1987, a revised version of the DSM’s third edition was released (DSM-III-R) (American Psychiatric Association, 1987), and proposed some changes to the previous DSM-III (American Psychiatric Association, 1980) definition of mental disorder. The following year, the well-known Wakefield works of 1992 (Wakefield, 1992a,b) appeared: the author discussed some previous approaches and then proposed a new definition based on his “harmful dysfunction” analysis. Wakefield proposed a “*hybrid account of disorder as harmful dysfunction, wherein dysfunction is a scientific and factual term based in evolutionary biology that refers to the failure of an internal mechanism to perform a natural function for which it was designed, and harmful is a value term referring to the consequences that occur to the person because of the dysfunction and are deemed negative by sociocultural standards*” (Wakefield, 1992a, p. 374). Wakefield’s proposal reinvigorated the debate on the definition of mental disorder and as a result a large number of scholars started to discuss his “harmful dysfunction” analysis, some in an extremely critical way, some endorsing it, some proposing minor or major changes, and others comparing it with different definitions and approaches.

Some years later, in 1994, the fourth edition of DSM was published and soon its revised edition was also released (American Psychiatric Association, 1994; American Psychiatric Association, 2000). More than ten years later, the fifth DSM edition (American Psychiatric Association, 2013) proposed a newer definition of mental disorder. In all the transitions between the various DSM versions, the debate on each proposed definition continued. Nowadays, we have reached the revised version of DSM-5 (DSM-5-TR) (American Psychiatric Association, 2022), and the 11th edition of ICD (World Health Organization, 2022). In this regard, although for practical reasons the definitions provided by the DSM-5 and DSM-5-TR currently appear to be the ones to have met more use worldwide, an active debate on the definition of mental disorder still persists, alongside the debate on other critical themes in psychopathology (like polythetic approach vs. hierarchical approach in the organization of diagnostic criteria, categorical vs. dimensional approaches, comorbidity vs. heterogeneity between and within mental disorders, and other critical themes). Two *general questions* emerged from this scenario: how has the debate on the definition of mental disorder developed during the last 50 years? And why is such a debate still open and unresolved? Keeping in mind all these aspects and with the purpose of addressing these *general questions*, in this paper we aim to review the works that have proposed, investigated, and discussed the definitions of “mental disorders” over the last 50 years, following the initial definitions proposed by Spitzer and Endicott (1978) and the DSM-III (American Psychiatric Association, 1980). More specifically, we aim at summarizing the debate on the definition of mental disorder and to discuss some of the proposed definitions based on the perspective of the proponent author(s) and/or of other author(s), according to the following *specific research questions:*

1. How did the author(s) of selected papers describe the concept of “Mental Disorder” and propose their own definition?
2. What other definition(s) did the author(s) of the selected papers report and discuss on the concept of “Mental Disorder”?
3. Did the author(s) of the selected papers refer to the definition(s) of mental disorders in the international diagnostic manual(s) (DSM or ICD)?

Considering the peculiarity of the topic, we chose the Scoping Review Approach (Peters et al., 2015; Tricco et al., 2018).

## Methods

This paper aimed at reviewing the works that have discussed and investigated the definition of “mental disorders,” starting from the first proposed definition by Spitzer and Endicott (1978) in sight of the third DSM edition (American Psychiatric Association, 1980). According to the “scoping review methodological approach,” we selected 64 papers and after careful examination we did both qualitative and quantitative analysis based on the described above research questions.

Table 1 shows the sorted paper that met the selection criteria.

**Table 1:** Description of the sorted papers.

References	Title	Country	Kind of paper
Kendell (1975)	The Concept of Disease and its Implications for Psychiatry	United Kingdom	Review
Frances et al. (1990)	DSM-IV: work in progress.	USA	Historical review
Stein (1991)	Philosophy and the DSM-III	USA	Report
Widiger and Trull (1991)	Diagnosis and clinical assessment	USA	Review
Wakefield (1992a)	The concept of mental disorder—on boundary between biological facts and social values	USA	Review
Wakefield (1992b)	Disorder as harmful dysfunction—a conceptual critique of DSM-III-R’s definition of mental disorder	USA	Review
Anderson and Khoo (1994)	Mental illness: diagnosis or value judgment?	United Kingdom	Clinical review
Lilienfeld and Marino (1995)	Mental disorder as a Roschian Concept: critique of Wakefield’s “harmful dysfunction” analysis	USA	Review
Follette and Houts (1996)	Models of scientific progress and progress and the role of theory in taxonomy development: a case study of the DSM	USA	Review
Widiger (1997)	The construct of mental disorder	USA	Response paper
Bergner (1997)	What is psychopathology? And so what?	USA	Narrative review
Wakefield (1998b)	The DSM’s theory-neutral nosology is scientifically progressive: Response to Follette and Houts (1996)	USA	Response paper
Houts and Follette (1998)	Mentalism, Mechanisms, and Medical Analogues: Reply to Wakefield (1998b)	USA	Response paper
Wakefield (1999a)	Philosophy of science and the progressiveness of the DSM’s theory-neutral nosology: response to Follette and Houts, part 1	USA	Response paper
Wakefield (1999b)	The concept of disorder as a foundation for the DSM’s theory-neutral nosology: response to Follette and Houts, part 2	USA	Response paper
Wakefield (1999c)	Evolutionary versus prototype analyses of the concept of disorder	USA	Response paper
Phillips (2000)	Conceptual models for psychiatry	USA	Review
Crowe (2000)	Constructing normality: A discourse analysis of the DSM-IV	New Zealand	Critical discourse analysis research
Houts (2001a)	The diagnostic and statistical manual’s new white coat and circularity of plausible dysfunctions: response to Wakefield, Part 1	USA	Response paper
Bolton (2001)	Problems in the definition of “mental disorder”	United Kingdom	Review
Wakefield (2001)	Evolutionary history versus current causal role in the definition of disorder: reply to McNally	USA	Response paper
Houts (2001b)	Harmful dysfunction and the search for value neutrality in the definition of mental disorder: response to Wakefield, part 2	USA	Response paper
Wakefield (2003)	Dysfunction as a factual component of disorder	USA	Response paper
Bolton (2004)	Values in the definition of mental disorder	United Kingdom	Review
Jablensky (2005)	Boundaries of mental disorders	Australia	Review
Berganza et al. (2005)	Concepts of disease: Their relevance for psychiatric diagnosis and classification	Guatemala / USA	Review
Wakefield (2006)	Are there relational disorders? a Harmful dysfunction perspective: a comment on the special section	USA	Response paper
Schwartz (2007a)	Defining Dysfunction: Natural Selection, Design, and Drawing a Line	USA	Review
Schwartz (2007b)	Distinguishing distress from disorder as psychological outcomes of stressful social arrangements: can we and should we?	USA	Response paper
Horwitz (2007)	Distinguishing distress from disorder as psychological outcomes of stressful social arrangements	USA	Narrative Review
Bolton (2007)	The useful of Wakefield’s definition for the diagnostic manuals	United Kingdom	Commentary
Wakefield (2007)	The concept of mental disorder: diagnostic implications of the harmful dysfunction analysis	USA	Review
Boysen (2008)	A reanalysis of relational disorders using Wakefield’s theory of harmful dysfunction	USA	Response paper
Frances et al. (2008)	Defining mental disorder when it really counts: DSM-IV-TR and SVP/SDP statutes	USA	Commentary
First and Frances (2008)	Issues for DSM-V: Unintended Consequences of Small Changes: The Case of Paraphilias	USA	Editorial
First and Wakefield (2010)	Defining mental disorder in DSM-V: a commentary on: What is a mental/psychiatric disorder? From DSM-IV to DSM-V	USA	Commentary
Stein et al. (2010)	What is a mental/psychiatric disorder? From DSM-IV to DSM-V	USA / UK	Editorial
Varga (2011)	Defining mental disorder. Exploring the “natural function” approach	Germany	Review
Malón (2012)	Pedophilia: A Diagnosis in Search of a Disorder	Spain	Review
Pierre (2012)	Mental illness and mental health: Is the glass half empty or half full?	USA	Review
Stier (2013)	Normative preconditions for the assessment of mental disorder	Germany	Review
Schramme (2013)	On the autonomy of the concept of disease in psychiatry	Germany	Review
Wakefield (2014)	The Biostatistical Theory Versus the Harmful Dysfunction Analysis, Part 1: Is Part-Dysfunction a Sufficient Condition for Medical Disorder?	USA	Response paper
Jacobs (2013)	Mental Disorder or “Normal Life Variation”? Why It Matters	USA	Review
Ulrich (2014)	Commentary to the articles of M. Stier (Normative preconditions for the assessment of mental disorder) and T. Schramme (On the autonomy of the concept of disease in psychiatry).	Switzerland	Commentary
Singh and Sinnott-Armstrong (2015)	The DSM-5 definition of mental disorder	USA	Essay
Troisi (2015)	The evolutionary diagnosis of mental disorder	Italy	Commentary
Surís et al. (2016)	The Evolution of the Classification of Psychiatric Disorders	USA	Concept paper
Schwartz (2017)	Progress in Defining Disease: Improved Approaches and Increased Impact	USA	Commentary
Horwitz (2017)	Social Context, Biology, and the Definition of Disorder	USA	Review
Gala and Laughon (2017)	Conceptualization of a mental disorder a Clinical perspective	USA	Clinical perspective
Bergner and Bunford (2017)	Mental disorder is a disability concept, not a behavioral one	USA	Research paper
Micoulaud-Franchi et al. (2017)	Keep calm and carry on: Mental disorder is not more “organic” than any other medical condition	France	Commentary
Telles-Correia et al. (2018)	Mental Disorder-The Need for an Accurate Definition	Portugal	Review
Amoretti and Lalumera (2019)	Harm should not be a necessary criterion for mental disorder: some reflections on the DSM-5 definition of mental disorder	Italy	Review
Bakker (2019)	A new conception and subsequent taxonomy of clinical psychological problems	Australia	Debate
Wakefield and Conrad (2020)	Harm as a Necessary Component of the Concept of Medical Disorder: Reply to Muckler and Taylor	USA / Belgium	Response paper
Wakefield (2020)	Addiction from the harmful dysfunction perspective: How there can be a mental disorder in a normal brain	USA	Response paper
Münch et al. (2020)	Should Behavior Harmful to Others Be a Sufficient Criterion of Mental Disorders? Conceptual Problems of the Diagnoses of Antisocial Personality Disorder and Pedophilic Disorder	Germany	Review
Nielsen and Ward (2020)	Mental Disorder as Both Natural and Normative: Developing the Normative Dimension of the 3e Conceptual Framework for Psychopathology	New Zealand	Review
Stein et al. (2021)	What is a mental disorder? An exemplar-focused approach	USA	Review
Gauld (2022)	From psychiatric kinds to harmful symptoms	France	Review
Telles-Correia (2022)	Values in mental and medical disorder concepts: Their presence is not the point, being aware of them is.	Portugal	Review
Biturajac and Jurjako (2022)	Reconsidering harm in psychiatric manuals within an explicationist framework.	Croatia	Review

### Protocol

The protocol was developed using the PRISMA-SCR’S scoping review methodological framework (Peters et al., 2015; Tricco et al., 2018). We conducted a literature review on the definition of mental disorder via Scopus, PubMed and Web of Science electronic databases, according to the following inclusion criteria: papers on the definition of Mental Disorder, written in English. All study designs were eligible, including those that utilized qualitative and quantitative methods, methodology or guidelines report. We excluded papers written in languages other than English.

### Information sources and search strategy

**Figure 1:**
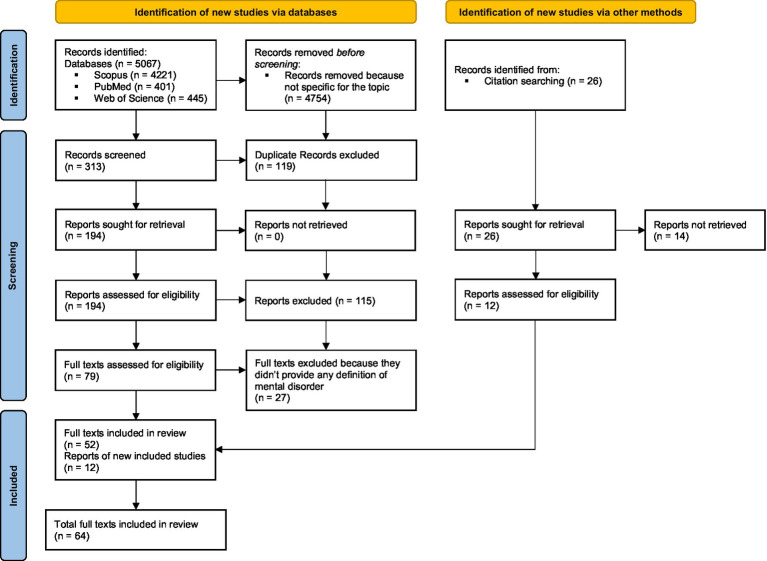
PRISMA checklist.

Literature searches were conducted by two authors (DRP and AM) via Scopus, PubMed, and Web of Science online databases, using the following search keywords: “mental disorder” combined with the “AND” Boolean operator and “meaning,” combined with the “OR” Boolean operator “definition.” Keywords were searched in the publication title or abstract. A total number of 5,067 records was found. The cited two authors (DRP and AM) independently reviewed the chosen references and decided to exclude some papers; a total of 313 abstract were selected. Duplicate references were also excluded. A total number of 194 papers was found. Finally, papers were analyzed with respect to their content, and papers that were not fully within the scope of this review were eliminated. A group of 79 full-text articles were considered. Starting from the references of the full-text articles derived from the literature review, 26 other papers were included. After reading the full-texts, a total of 64 papers were then considered for the final analysis (Figure 1).

### Geographical distribution

The geographical distribution of the sorted papers suggested a prevalence of interest in the USA (Frances et al., 1990, 2008; Stein, 1991; Widiger and Trull, 1991; Wakefield, 1992a,b, 1998a, 1999a,b,c, 2001, 2003, 2006, 2007, 2014, 2020; Lilienfeld and Marino, 1995; Follette and Houts, 1996; Bergner, 1997; Widiger, 1997; Houts and Follette, 1998; Phillips, 2000; Houts, 2001a,b; Horwitz, 2007, 2017; Schwartz, 2007a,b, 2017; Boysen, 2008; First and Frances, 2008; First and Wakefield, 2010; Stein et al., 2010, 2021; Jacobs, 2013; Singh and Sinnott-Armstrong, 2015; Surís et al., 2016; Bergner and Bunford, 2017; Gala and Laughon, 2017; Wakefield and Conrad, 2020). Some authors from Europe have also addressed the topic (UK, Italy, Germany, Belgium, Spain, Switzerland, France, Portugal, Greece, Croatia) (Kendell, 1975; Anderson and Khoo, 1994; Bolton, 2001; Bolton, 2004, 2007; Malón, 2012; Pierre, 2012; Stier, 2013; Schramme, 2013; Ulrich, 2014; Troisi, 2015; Micoulaud-Franchi et al., 2017; Telles-Correia et al., 2018; Amoretti and Lalumera, 2019; Münch et al., 2020; Gauld, 2022; Telles-Correia, 2022; Biturajac and Jurjako, 2022). Only a few authors from other countries, like New Zealand and Australia focused on the topic of this review (Crowe, 2000; Jablensky, 2005; Bakker, 2019; Nielsen and Ward, 2020).

### Kind of papers

The selected papers are mainly editorials, commentaries, viewpoint papers, perspective papers, articles in special issues or report (Kendell, 1975; Frances et al., 1990, 2008; Stein, 1991; Widiger and Trull, 1991; Wakefield, 1992a,b, 1998a, 1999a,b,c, 2001, 2003, 2006, 2007, 2014, 2020; Anderson and Khoo, 1994; Lilienfeld and Marino, 1995; Follette and Houts, 1996; Bergner, 1997; Widiger, 1997; Houts and Follette, 1998; Crowe, 2000; Phillips, 2000; Bolton, 2001, 2004, 2007; Houts, 2001a,b; Berganza et al., 2005; Jablensky, 2005; Horwitz, 2007, 2017; Schwartz, 2007a,b, 2017; Boysen, 2008; First and Frances, 2008; First and Wakefield, 2010; Stein et al., 2010, 2021; Varga, 2011; Malón, 2012; Pierre, 2012; Jacobs, 2013; Schramme, 2013; Stier, 2013; Ulrich, 2014; Singh and Sinnott-Armstrong, 2015; Troisi, 2015; Surís et al., 2016; Gala and Laughon, 2017; Micoulaud-Franchi et al., 2017; Telles-Correia et al., 2018; Amoretti and Lalumera, 2019; Bakker, 2019; Münch et al., 2020; Nielsen and Ward, 2020; Wakefield and Conrad, 2020; Biturajac and Jurjako, 2022; Gauld, 2022; Telles-Correia, 2022) and only one research article focused on the themes of this scoping review (Bergner and Bunford, 2017).

## Results

### How did the author(s) of selected papers describe the concept of “mental disorder” and propose his/her own definition?

Out of the 64 sorted papers, only 30 papers directly proposed a specific definition while the other 34 did not propose any of their own. If we look deeper at those 30 papers we may notice that a considerable amount are written by Jerome Wakefield to propose various slight changes to his “harmful dysfunction” analysis (Wakefield, 1992a,b, 1998a, 1999a,b,c, 2001, 2003, 2006, 2007, 2014, 2020; First and Wakefield, 2010). Anyway, if we count Wakefield’s analysis once, the number of proposed definitions reduces to about 15 definitions (if we also count some highly relevant statements about definitional issues) (Widiger and Trull, 1991; Wakefield, 1992a; Lilienfeld and Marino, 1995; Horwitz, 2007, 2017; Schwartz, 2007a; First and Wakefield, 2010; Stein et al., 2010; Malón, 2012; Schramme, 2013; Stier, 2013; Troisi, 2015; Gala and Laughon, 2017; Nielsen and Ward, 2020; Gauld, 2022). If we consider all the papers that have endorsed a specific definition or some particular approach about the definition of mental disorder the number of relevant papers rises to 47. By saying “papers that have endorsed some particular approach about the definition” we refer to those papers that although did not propose a specific literal definition, still endorsed a view or approach about relevant definitional issues. In our analysis such definitions and approaches can be grouped and briefly described as follows: dysfunction-requiring definitions, distress or disability-requiring definitions, dysfunction and distress or disability-requiring definitions, statistics-based definitions, Roschian concept approaches, integrations between definitions and add-ons, and finally critical approaches to the concept mental disorder (see Table 2 for all the references). We are well aware that the identified groups of definition could not be totally exhaustive and could not fully represent the complexity of the long debate on the definition of mental disorder, anyway we hope that such schematization can contribute to provide an overview of the debate.

**Table 2:** Groups of definitions and approaches with relative description and references.

Definition groups	Description	References
Dysfunction-requiring definitions	We included here the authors who considered the concept of “dysfunction” to be the essential part of mental disorder and made it a *conditio sine qua non*. Note that the authors eventually conceptualized dysfunction itself in different ways.	Boorse (1977), Klein (1978), Boorse (1997), Matthewson and Griffiths (2017), Nielsen and Ward (2020)
Distress or disability-requiring definitions	We included here the authors who considered the negative consequences on adaptation as well as individual discomfort, as the central features of mental disorder, rather than dysfunction.	Woodruff et al. (1974), Ossorio (1985), Widiger and Trull (1991), Widiger (1997), Bergner (1997), Megone (1998), Bergner and Bunford (2017), Gala and Laughon (2017), Troisi (2015), Telles-Correia et al. (2018), Gauld (2022)
Dysfunction and distress or disability-requiring definitions	We included here the papers who argued mental disorder to require both a dysfunction and negative consequences such as distress and/or functional impairment.	Spitzer and Endicott (1978); Wakefield (1992a,b, 1999a, 2001, 2003, 2006, 2007, 2014, 2020), First and Wakefield (2010); Wakefield and First (2013), Wakefield and Conrad (2020); Stein et al. (2010, 2021)
Statistics-based definitions	We included here the definitions that discussed the concept of mental disorder referring to “levels of functioning” and to distributions of functioning, in which certain cut offs determine the line between pathology and non-pathology.	Cohen (1943), Scadding (1967), Kendell (1975), Boorse (1977), Boorse (1997)
Roschian concept approaches	We included in this group all the considerations made by authors who argued mental disorder to be a prototype concept.	Frances et al. (1990), Lilienfeld and Marino (1995), Gala and Laughon (2017), Gauld (2022)
Integrations between definitions and add-ons	We included here the authors who proposed some kind of integration between more definitions and/or proposed add-ons to previous definitions.	Schwartz, (2007a, 2017) and Malón (2012)
Critical approaches to mental disorder definition	We included here the authors who skeptically discussed the concept of mental disorder and/or argued mental disorder to be a value-laden and culturally driven concept.	Szasz (1960, 1974, 1994), Goffman (1968), Crowe (2000), Cooper (2002), Jacobs (2013), Horwitz (2017)

We included in the dysfunction-requiring group, all those definitions of mental disorder and/or approaches to definition that have considered the existence of a dysfunction in the individual as the fundamental requirement for mental disorder. However, it is better to clarify that the included papers have often conceptualized dysfunction in different ways, such in the case of Wakefield’s “harmful dysfunction” analysis which considers dysfunction to be *“the failure of an internal mechanism to perform a natural function for which it was designed”* (Wakefield, 1992a, p. 374), and the case of Nielsen and Ward (2020) according to whom *“what counts as mentally dysfunctional is any set of behaviors (inclusive of cognition, perception—anything the organism does) performed by an organism that significantly violates its own functional norms, in that it is acting counter to its own self-maintenance and adaption needs”* (Nielsen and Ward, 2020, p. 22). It is also remarkable that while some of the authors referred to the concept of dysfunction relating it to some kind of inner organismic malfunction (we have included these authors in the dysfunction-requiring group), other authors seemed to equate it, or to use the term in an interchangeable way with the idea of functional impairment and disability (we have included these authors in the distress or disability-requiring group instead). In this regard, we included in the distress or disability-requiring group all the papers that considered the negative consequences (such as harm, distress, disability, maladaptation and functional impairment) as the main feature of mental disorder, rather than the presence of a dysfunction of some sort. This is the case of authors such as Bergner and Bunford (2017) who endorsed a disability conception of mental disorder and described the several advantages of using this approach to definition. In the following group, namely “dysfunction and distress or disability-requiring definitions” we included the papers that defined mental disorder based on the compresence of both dysfunction and a series of negative consequences in the individual such as distress and disability, making both features necessary for disorder. Are included in this group Wakefield’s “harmful dysfunction” analysis (Wakefield, 1992a,b) as well as all the definitions of mental disorder provided by the DSM since its third edition (American Psychiatric Association, 1980, 1987, 1994, 2000, 2013, 2022), and some other authors (see Table 2). We regarded as “statistics-based definitions” all definitions and/or approaches that drew the line between pathology and non-pathology based on a “level of functioning” criteria in a statistical distribution, and on the deviation from norm (mean functioning) in a given reference class, such as in the case of Cohen (1943) and Boorse (1977). We also considered it useful for this analysis to report the “Roschian concept” approach to the definition of mental disorder, representing mental disorder as a prototype concept with no clear boundary or universal defining features. Some of the included papers directly referred to Eleanor Rosch prototype theory (Rosch, 1973; Rosch and Mervis, 1975) such in the case of Lilienfeld and Marino (1995), who criticizing Wakefield’s “harmful dysfunction” analysis (Wakefield, 1992a,b), argued that mental disorder can be better understood as a Roschian prototype concept, without clear boundaries or stable universal features. Following the same direction, Gala and Laughon (2017) declared to be *“worried that the degree of heterogeneity across mental disorders makes the search for a single essentialist definition that will encompass all disorders akin to the search for the Holy Grail”* (p. 41), and although agreed that disability is a core feature of mental disorder, they endorsed a prototype definition of disorder. We included a few papers that did not really fit in any of the aforementioned groups in a separate category, containing the papers that even if did not propose a definition of their own, proposed some kind of integration between different conceptions or add-ons to previous definitions. This is the case of Malón (2012), who, highlighting that some disorders do not necessarily entail distress or disability in the individual, proposed the notion of “dangerous dysfunction,” to include in the definition, disorders such as the pedophilic disorder (which is often reported as a counterexample to approaches to the definition of disorder based on the negative consequences in the affected individual). We also included in the same group Schwartz’s work (Schwartz, 2007a), in which the author proposed an interesting resolution of the “line-drawing problem” through a model of mental disorder that integrates the dysfunction-requiring account, the distress or disability requirement, and the statistical approach. Finally we addressed more radical arguments mainly proposed by authors from the anti-psychiatry perspective (Szasz, 1960, 1974, 1994), or by some other papers arguing the concept of mental disorder to be value laden and culturally driven (Goffman, 1968; Cooper, 2002; Crowe, 2000). We included in this last group also Jacobs (2013) work, who strongly criticized DSM-5 (American Psychiatric Association, 2013) definition of mental disorder and argued for a different and more person-oriented approach, as well as Horwitz (2017) considerations about the relevance of context and cultural norms in the evaluation of mental disorders.

### What other definition(s) did the author(s) of selected papers report and discuss on the concept of “mental disorder” (if any)?

In order to reply to this specific research question, we analyzed the sorted paper searching for quotations and/or discussions of previous and current mental disorder definitions and approaches proposed by other authors. We found that most of the papers discussed or cited mental disorder definitions and approaches proposed by other scholars (Kendell, 1975; Frances et al., 1990; Stein, 1991; Wakefield, 1992a,b, 1998a, 1999c; Anderson and Khoo, 1994; Lilienfeld and Marino, 1995; Follette and Houts, 1996; Bergner, 1997; Houts and Follette, 1998; Crowe, 2000; Phillips, 2000; Houts, 2001a; Bolton, 2004; Horwitz, 2007, 2017; Schwartz, 2007a, 2017; Stein et al., 2010, 2021; Malón, 2012; Pierre, 2012; Schramme, 2013; Stier, 2013; Singh and Sinnott-Armstrong, 2015; Troisi, 2015; Surís et al., 2016; Bergner and Bunford, 2017; Micoulaud-Franchi et al., 2017; Telles-Correia et al., 2018; Biturajac and Jurjako, 2022; Telles-Correia, 2022).

Some authors focused mainly on one definition of mental disorders (Anderson and Khoo, 1994; Houts and Follette, 1998; Crowe, 2000; Bolton, 2004; Stein et al., 2010, 2021; Malón, 2012; Pierre, 2012; Schramme, 2013; Ulrich, 2014; Surís et al., 2016; Micoulaud-Franchi et al., 2017; Schwartz, 2017), while other authors discussed mainly on more definition and/or theoretical frameworks (some papers discussed six or seven different definitions/approaches) (Kendell, 1975; Stein, 1991; Wakefield, 1992a,b; Follette and Houts, 1996; Bergner, 1997; Phillips, 2000; Houts, 2001b; Schwartz, 2007a; Stier, 2013; Troisi, 2015; Bergner and Bunford, 2017; Telles-Correia et al., 2018; Biturajac and Jurjako, 2022; Telles-Correia, 2022). In this regard, Wakefield’s definition (Wakefield, 1992a,b) together with Spitzer and Endicott (1978) definition and the ones proposed in the various DSM editions (American Psychiatric Association, 1980, 1987, 1994, 2000, 2013, 2022) had a central role.

### Did the author(s) of the selected papers refer to the definition(s) of mental disorders in the international diagnostic manual(s) (DSM or ICD)?

Aiming at replying to this specific research question, we analyzed the sorted paper searching for quotations and/or discussions of the definition(s) of mental disorders in the international diagnostic manual(s) (DSM or ICD). Most of the works mentioned the definitions proposed by the international diagnostic manuals (Frances et al., 1990, 2008; Stein, 1991; Wakefield, 1992a,b, 1998a, 1999a,b,c, 2003, 2014; Lilienfeld and Marino, 1995; Follette and Houts, 1996; Bergner, 1997; Houts and Follette, 1998; Crowe, 2000; Phillips, 2000; Bolton, 2001, 2007; Houts, 2001a; Berganza et al., 2005; Jablensky, 2005; Horwitz, 2007, 2017; Schwartz, 2007b; Bolton, 2008; First and Frances, 2008; First and Wakefield, 2010; Stein et al., 2010, 2021; Malón, 2012; Pierre, 2012; Jacobs, 2013; Schramme, 2013; Singh and Sinnott-Armstrong, 2015; Troisi, 2015; Surís et al., 2016; Bergner and Bunford, 2017; Gala and Laughon, 2017; Micoulaud-Franchi et al., 2017; Telles-Correia et al., 2018; Amoretti and Lalumera, 2019; Bakker, 2019; Münch et al., 2020; Wakefield and Conrad, 2020; Biturajac and Jurjako, 2022) they often cited the various editions of the DSM (American Psychiatric Association, 1952, 1968, 1980, 1987, 1994, 2000, 2013, 2022), although mostly in a critical way, while the ICD was significantly less cited (Frances et al., 1990; Follette and Houts, 1996; Wakefield, 1998a, 1999a, 2014; Phillips, 2000; Bolton, 2001, 2007; Houts, 2001a; Berganza et al., 2005; Jablensky, 2005; Schramme, 2013; Singh and Sinnott-Armstrong, 2015; Bakker, 2019; Münch et al., 2020; Stein et al., 2021). Some authors highlighted and discussed specific points about the DSM definitions, such as the concept of “clinically significant” (Wakefield, 1992a, 2014; Bolton, 2001; Jablensky, 2005), the concepts of “harm,” “distress” and “disability” (Wakefield, 1992a,b; Berganza et al., 2005; Horwitz, 2007, 2017; Schwartz, 2007b; First and Frances, 2008; Malón, 2012; Troisi, 2015; Telles-Correia et al., 2018; Amoretti and Lalumera, 2019; Münch et al., 2020; Biturajac and Jurjako, 2022), and the “dysfunction in the individual” requirement (Wakefield, 1992a,b; Follette and Houts, 1996; Bolton, 2001; Houts, 2001a; Horwitz, 2007; Schwartz, 2007b; Troisi, 2015; Bergner and Bunford, 2017; Telles-Correia et al., 2018). Another frequently discussed point regarded the challenge of clearly distinguishing between “socially deviant behavior” and “mental disorder” (Frances et al., 2008; First and Frances, 2008; Malón, 2012; Münch et al., 2020; Biturajac and Jurjako, 2022).

## Discussion

The purpose of this review is to summarize the debate concerning the definition of mental disorder over the last 50 years. Taking into consideration the sorted papers, we addressed two *general questions* and three *specific research questions* to discuss this topic in a deeper way.

With regard to the *first general question*, the selected papers described and analyzed a complex and intense debate on the definition of mental disorder that started soon after the first definition proposed by Spitzer and Endicott (1978), continued over the years, and it is still going on. Moving the focus toward the *three more specific research questions*, the *first specific research question* highlighted that only few authors directly proposed one or more than one personal definitions. In this regard we showed an overview of the main approaches to the definition of mental disorder that emerged (dysfunction-requiring definitions, distress or disability-requiring definitions, dysfunction and distress or disability-requiring definitions, statistics-based definitions, roschian concept approaches, integrations between definitions and add-ons, and skeptic or critical approaches to the concept mental disorder). Then, addressing the *second specific research question*, we found that most of the authors discussed, analyzed, and/or criticized definitions proposed by other author(s), showing the complexity of the debate. Wakefield’s “harmful dysfunction” analysis (Wakefield, 1992a,b) together with Spitzer and Endicott (1978) definition and the definitions proposed in the various DSM editions (American Psychiatric Association, 1980, 1987, 1994, 2000, 2013, 2022) had a central role, and undoubtedly are the ones to have been overall most cited and discussed. Concerning the third specific research question, most of the sorted papers (n = 64) mentioned definitions from international diagnostic manuals, with the DSM being referenced more frequently than the ICD. We hypothesized that such a disparity of citation between the two main international diagnostic manuals could be the result of the high number of authors from the USA who discussed the topic of this review (42 of the sorted papers were from the USA compared to 22 which came from different countries). Lastly, concerning the *second general question*, data from the selected papers indicate that the debate on the definition of mental disorder is still open and vital, as demonstrated by the presence of recent articles on the topic of this scoping review (Wakefield and Conrad, 2020; Wakefield, 2020; Münch et al., 2020; Nielsen and Ward, 2020; Stein et al., 2021; Gauld, 2022; Telles-Correia, 2022; Biturajac and Jurjako, 2022) or other recent papers on similar topics (Richter and Dixon, 2023). This also shows that currently, even after a so prolonged debate, there still is not at time a unique agreed upon definition of mental disorder. One reason why the debate is still open may be related to the two main paths followed by the authors of the papers: the first focusing on the definitions of mental disorder directly proposed by the authors, along with the proposed approaches addressing specific definitional issues, and the second focusing instead on the evolution of the DSM definitions across its various editions (American Psychiatric Association, 1980, 1987, 1994, 2000, 2013, 2022). In this regard, even if the mentioned paths mostly proceed in a separate way, some points of contact can be identified. The first point of contact surely emerges in Wakefield’s “harmful dysfunction” analysis (Wakefield, 1992a,b) in which the author discussed DSM-III-R’s (American Psychiatric Association, 1987) definition of mental disorder and argued that such a definition contains two fundamental principles: 1) a disorder is a condition that has negative consequences for the person, and 2) a disorder is a dysfunction. Anyway, according to Wakefield, failing to indicate what a dysfunction effectively is, DSM-III-R’s (American Psychiatric Association, 1987) definition fails to validly distinguish disorders from non-disorders. Wakefield’s two papers of 1992 (Wakefield, 1992a,b) then attracted much interest and had the result of further stimulating the debate about the definition of mental disorder, influencing both of the two paths described above. A second point of contact emerges from the debate about those conditions regarded as disorders that do not necessarily entail distress and/or disability. In this regard, the definition of mental disorder provided in the various editions of DSM changed numerous times, and made slight adaptations to adapt to the current debate. This is the case of the shift from the idea of mental disorder as a syndrome or pattern that is “associated” with distress or disability in DSM-III-R, DSM-IV and DSM-IV-TR (American Psychiatric Association, 1987, 1994, 2000) to the idea of disorder as “*usually* associated” to distress or disability (as in DSM-5 and DSM-5-TR; American Psychiatric Association, 2013, American Psychiatric Association, 2022). This apparently small but highly relevant change was pivotal in order to include in the category of mental disorder some conditions that does not necessarily entail distress and/or disability (such as the pedophilic disorder and the antisocial personality disorder) (Malón, 2012; Münch et al., 2020). In a similar way, the complexity of the debate emerges in other highly discussed matters such as “the dysfunction in the individual” requirement (Wakefield, 1992a,b; Follette and Houts, 1996; Bolton, 2001; Houts, 2001a; Horwitz, 2007; Schwartz, 2007b; Troisi, 2015; Bergner and Bunford, 2017; Telles-Correia et al., 2018), the definition of the concept of “dysfunction” itself, and the challenge of clearly distinguishing between “socially deviant behavior” and “mental disorder” (Frances et al., 2008; First and Frances, 2008; Malón, 2012; Münch et al., 2020; Biturajac and Jurjako, 2022). Therefore, the two paths and their important point of contact contributed to the debate on the definition of mental disorder.

## Conclusions and limitations

This scoping review aimed to summarize and discuss the debate on the definitions of mental disorder in the last 50 years. The selected papers gave us the opportunity to look in a deeper way at the definitions of mental disorder that, in the last 50 years, have been proposed and discussed starting from the first definition proposed by Spitzer and Endicott (1978).

Even if there have been some changes between the various definitions of mental disorder proposed and discussed, there are some main points of contact between all of them and the Spitzer and Endicott (1978) definition itself: first, a description of signs and symptoms on various levels of psychological functioning; second, a reference to etiology and/or dysfunction on various levels; third, a reference to the consequences of mental disorder itself on the individual’s life, according to various levels and/or domains of life. The same structure is also present in the definitions of mental disorder proposed in the international diagnostic manuals (American Psychiatric Association, 1980, 1987, 1994, 2000, 2013, 2022). Anyway, even after having highlighted these three main points of contact between the various proposed definitions of mental disorder, some relevant divergences are still identifiable. In this regard, the terminologies and semantics used in each definition changed a lot, with such changes especially concerning: the levels of psychological functioning (from behavioral and psychological, to emotional/relational/cognitive); the etiology and causes of mental disorder (the proposed definitions range from explicit reference to some specific cause of mental disorder, to vague reference to presumably multilevel causal models); and the overall and specific consequences on individual’s life (ranging from disability, maladjustment and functioning, each of them may merit a focus on its semantic/definition) (see for example Masala and Petretto, 2008 for some definitions of disability and functioning).

Anyway, the debate on the definition of “mental disorder” is still ongoing and a unique shared definition is not yet present; even if the definitions proposed by the international diagnostic manuals (especially by the DSM, and more specifically the ones proposed by DSM-5 and DSM-5-TR (American Psychiatric Association, 2013, 2022) constitute undeniable landmarks, such definitions resulted to be more the outcome of various discussions, than the fruit of a currently shared consensus. We believe that there is still a lot of work to be done in this field, and we agree with Kraepelin on the idea that everything can change to better study “psychopathology” and to address specific issues. Taking into consideration the perspective of clinical psychology, which aims to address the needs of the individual and to support the individual’s life project, we may ask if these older and/or newer definitions are really useful. Are the current proposed definitions of mental disorder useful more for diagnosis than they are for intervention? Are the current proposed definitions of mental disorder mainly based on the polythetic diagnostic approach, just as the one on which the diagnostic criteria are currently based? Do they provide a clear description of the “core signs” and “core symptoms” underlying mental disorders? Can the current proposed mental disorder definitions take account of the consistency/continuity of the psychopathological features/traits during an individual’s lifespan? Can the current proposed definitions of mental disorder take account of the homotypic or heterotypic continuity of an individual’s psychopathological features throughout different life stages? Can the current proposed definitions describe the homotypic or heterotypic continuity among different individuals who are at the same stage in life? Are the current proposed definitions of mental disorder useful for specific interventions?

Further research is thus needed, along two main different directions: looking at the past, and looking at the future. Looking at the future, we may refer to the promising multilevel and multi causal approaches to psychopathology, like the one based on the study of the developmental trajectories of signs and symptoms, and of behavior features/traits, along the various phases of each individual’s life cycle; as well as those approaches based on the need to integrate the study of the cited individual’s features with the study of the effects of environmental factors (e.g., social and relational factors, and protective or risks factors) (Cicchetti and Sroufe, 2000; Cicchetti, 2016; Venta et al., 2021).

Bearing in mind all those aspects, without any doubts, the construct of mental disorder and its shared definition remains a critical theme in psychopathology.
